# The Effect of Educational Intervention on Adherence to Treatment Recommendations and Quality of Life in Patients With Liver Cirrhosis

**DOI:** 10.7759/cureus.81737

**Published:** 2025-04-04

**Authors:** Aikaterini Oikonomou, Nikolaos Fotos, Anastasia A Chatziefstratiou, Konstantinos Giakoumidakis, Ioannis Elefsiniotis, Hero Brokalaki

**Affiliations:** 1 Department of Nursing, School of Health Sciences, National and Kapodistrian University of Athens, Athens, GRC; 2 Department of Nursing, School of Health Sciences, Hellenic Mediterranean University, Heraklion, GRC; 3 Academic Department of Internal Medicine, General Oncology Hospital of Kifisia "Agioi Anargyroi" National and Kapodistrian University of Athens, Athens, GRC

**Keywords:** educational intervention, liver cirrhosis, patient adherence, quality of life, treatment recommendations

## Abstract

Introduction

Liver cirrhosis (LC) is a chronic disease with serious complications affecting adversely patients' quality of life (QoL), leading to a significant burden on the healthcare system. Effective management of LC involves both treating the underlying etiology to delay disease progression and addressing long-term complications. Insufficient adherence of patients to treatment recommendations is considered a major factor of ineffective disease management.

Methods

This is a controlled interventional prospective study, involving cirrhotic patients who were followed up at the outpatient hepatology department of a general hospital in Athens from January 2015 to September 2018. The educational intervention consisted of one session supported by a nurse along with a specific information leaflet. Data were collected at patients’ initial evaluation and subsequently at thee and six months. Adherence was estimated by the A-14 scale and QoL by the Chronic Liver Disease Questionnaire (CLDQ). Statistical analysis was performed by the use of the SPSS 22.0 statistical program (IBM Corp., Armonk, NY, USA). The level of statistical significance was set at 0.05.

Results

A total of 125 patients participated in the study of whom 65 (52%) were included in the intervention group and 60 (48%) in the control group. Patients’ mean ± standard deviation age was 66.5±11.8 and 64.2±13.7 years in the intervention and control group, respectively. The educational intervention led to a statistically significant improvement in adherence to treatment recommendations, and this effect was maintained during the 6-month follow-up period (p<0.001). Additionally, the educational intervention improved the overall QoL (p<0.001) and reduced the proportion of patients with at least one readmission as well as the total number of readmissions (p<0.001) during the 6-month follow-up period. Multivariate analysis showed that the effect of the educational intervention on patients’ adherence to treatment recommendations and QoL, was independent of patients’ demographic and clinical characteristics.

Conclusions

Ongoing education is an important nursing intervention for improving both LC patients' adherence to treatment recommendations and their QoL.

## Introduction

Liver cirrhosis (LC) is an irreversible chronic liver disorder caused by many etiologies and characterized by fibrosis, destruction of the normal liver architecture, and formation of structurally abnormal regenerative nodules. The most common causes of cirrhosis are alcoholic and non-alcoholic fatty liver disease, viral hepatitis (B or C), primary biliary cholangitis, autoimmune or cryptogenic hepatitis, metabolic inherited disorders, and drug-induced hepatotoxicity.

Disease progression and healthy tissue replacement by fibrotic tissue are associated with early clinical manifestations such as fatigue, anorexia, weight loss, nausea, and abdominal pain or distension. Common complications of LC include portal hypertension leading to ascites, spontaneous bacterial peritonitis, variceal bleeding, cirrhotic hydrothorax, spontaneous bacterial pleural effusion, hepatic encephalopathy, and hepatopulmonary syndrome [1].

Liver cirrhosis is diagnosed histologically through liver biopsy and/or elastography (FibroScan), a new non-invasive, imaging technique. The Child-Pugh classification system allows the quantification of hepatic impairment in patients with LC based on clinical examination and common laboratory tests [2, 3]. The elements of this classification system include the presence of hepatic encephalopathy and ascites and the measurement of serum bilirubin, albumin, and prothrombin time values [4].

Etiological treatment of LC can delay the progression of the disease and reduce the risk of developing hepatocellular carcinoma (HCC) [5]. On the other hand, several common medications are used to treat complications of cirrhosis. Diuretics reduce fluid retention and ascites, while laxatives are usually prescribed in patients with hepatic encephalopathy. Non-selective as well as combined alpha- and beta-blockers are given to prevent complications of cirrhosis by reducing the portal hypertension.

Regardless of treatment effectiveness, quality of life (QoL) in patients with LC remains low since the stage of cirrhosis and severity of symptoms have a strong impact on daily activities and emotional functionality [6]. Patients with LC are required to significantly modify their lifestyle in order to manage several clinical manifestations of the disease and delay the onset of serious complications [7].

However, adherence of LC patients to medications and prevention programs, such as annual vaccination and screening for HCC (18-28% of patients), has been found to be insufficient [8, 9]. Strategies to enhance patients’ adherence, such as education, seem to contribute to effective self-management of the disease, prevent further decompensation and improve QoL [10].

The aim of the study was to evaluate the effect of education on adherence to treatment recommendations in patients with LC by comparing adherence and QoL between LC patients who participated in the educational process versus LC patients who received the usual care.

## Materials and methods

Study design

In this 6-month controlled interventional prospective study, LC consecutive patients were initially screened and enrolled after convenience sampling at the Hepatology Outpatient Clinic of a general hospital in Athens from January 2015 to September 2018. All recruited patients met the international classification criteria for cirrhosis based on Fibroscan or non-invasive biomarkers and according to physicians’ judgment.

The inclusion criteria of LC patients were: 1) liver cirrhosis of all causes, 2) at least one month of LC treatment, 3) Child-Pugh stage A or B, 4) age ≥18 years, 5) adequate knowledge of the Greek language, 6) informed written consent of participation to the study. The exclusion criteria included the following: 1) known cognitive deficits, disturbances of memory, and neurological diseases, 2) cancer patients with a performance status >1, according to the ECOG scale, 3) psychiatric diseases, 4) active drug use one month before initial estimation, 5) Grade III or IV hepatic encephalopathy/Child-Pugh stage C, 6) HIV co-infection, 7) end-stage chronic renal failure, 8) Class II-IV heart failure (according to New York Heart Association Functional Classification).

Patients were randomized following the simple randomization strategy into two groups: intervention and control group. Patients in the intervention group were subject to a single, 30-minute educational session provided by a specialist nurse at baseline, and a relevant leaflet was delivered to each patient at the end of this session (supplementary material/Appendix B). In the control group, no educational intervention was performed and patients received directly the usual care by the treating physician.

All patients were evaluated at baseline, three and six months after recruitment, and the two groups were compared in terms of adherence and QoL. Patients’ adherence to medication was estimated with the A-14 scale [11], while adherence to non-pharmaceutical recommendations was evaluated with a new tool designed by the researchers (supplementary material/Appendix A). This ten-item Likert scale demonstrated reliability and validity for use in the Greek population (Cronbach’s α = 0.79). The overall score ranges from 10 to 50, with higher scores indicating greater adherence. Quality of life was measured with the Greek version of the Questionnaire for Chronic Liver Disease-CLDQ [12].

The study was approved by the Ethics and Deontology Committee of the Department of Nursing, School of Health Sciences of National and Kapodistrian University of Athens (Protocol number: 974) and the Scientific Council of the General Hospital of Athens in which the study was conducted (protocol number: 18310). Oral permission was also given by the head physician and the head nurse of the Hepatology department who were informed about the nature and purpose of the study. Full confidentiality and anonymity were maintained and the investigation was carried out in accordance with the Helsinki Declaration of 1975, as revised in 2013. Written informed consent was obtained from all individual participants included in the study.

Statistical analysis

Mean values, standard deviations (SD) and median and interquartile ranges were used to describe the quantitative variables. Absolute (n) and relative (%) frequencies were used to describe qualitative variables. Pearson’s x^2^ test or Fisher’s exact test was used to compare ratios, where necessary. The McNemar test was used to compare the ratios before and after the intervention. Student’s t-test or the non-parametric Mann-Whitney criterion was used to compare quantitative variables between the two groups. The paired t-test was used to compare the factors between the measurements. A mixed-effects model of repeated-measurements analysis of variance (ANOVA) was used to evaluate the changes observed in study variables among the different groups over the follow-up period. The rate of change in patients’ adherence based on the A-14 scale at follow-up was checked using logistic mixed models, which resulted in relative ratios (Odds ratio) with 95% confidence intervals (95%). The internal reliability of the A-14 scale was tested using the Cronbach’s α coefficient. The statistical significance level was set to p=0.05. All statistics were conducted using the SPSS 22.0 software package (IBM Corp., Armonk, NY, USA).

The sample size was calculated a priori, using the G*power 3.1.9.7 software by applying the following settings: effect size=0.3, a level=0.05, power=0.8, df=1 (for chi-square family tests). The calculated total sample size was 88 patients.

## Results

Main demographic and clinical features

One hundred thirty-four patients with LC were initially evaluated; nine did not meet the inclusion criteria and were excluded because they had a co-existing chronic life-threatening illness, such as HIV and HCC [3 (2.24%)], Grade III or IV hepatic encephalopathy [3 (2.24%)], past medical history of psychiatric disorder (bipolar disorder) [1 (0.75%)] or they did not agree to participate in the study [2 (1.49%)]. Finally, 125 LC patients were included, matched according to gender, age, education level and cirrhosis etiology, and classified into two groups: 65 (52%) in the intervention group and 60 (48%) in the control group. The main demographic and clinical features of the patients included in the study are shown in Table 1.

**Table 1:** Characteristics of cirrhotic patients ^†^p-values represent comparisons between the two groups in each parameter separately (p-values less than 0.05 were considered statistically significant) ^+^Statistic value of the test (degrees of freedom) ^*^Chi-square test, ^**^Student’s t-test, ^***^Fisher’s exact test

Patients’ Characteristics		Intervention Group (n=65)	Control Group (n=60)	Test value (df)^+^	p-value^†^
Gender	Male	42 (64.6%)	36 (60.0%)	0.28 (1)	0.595*
	Female	23 (35.4%)	24 (40.0%)		
Age (mean ± standard deviation)		66.5 (11.8%)	64.2 (13.7%)	-0.99 (123)	0.323**
Education level	Low	20 (30.85%)	20 (33.3%)	0.18 (2)	0.913*
	Middle	29 (44.6%)	27 (45.0%)		
	High	16 (24.6%)	13 (21.7%)		
Living conditions	Alone	9 (13.8%)	10 (16.7%)	0.19 (1)	0.661*
	With family/other	56 (86.2%)	50 (83.3%)		
Professional status	Working	10 (15.4%)	17 (28.3%)	6.20 (5)	0.291***
	Unemployed	12 (18.5%)	10 (16.7%)		
	Retired	34 (52.3%)	29 (48.3%)		
	Disability	6 (9.2%)	2 (3.3%)		
	Household	3 (4.6%)	1 (1.7%)		
	None of the above	0 (0.0%)	1 (1.7%)		
Monthly family income	<600€	26 (40.0%)	23 (38.3%)	0.04 (2)	1.000***
	600-1000€	35 (53.8%)	33 (55.0%)		
	>1000€	4 (6.2%)	4 (6.7%)		
Comorbidities	No	31 (47.7%)	34 (56.7%)	1.01 (1)	0.316*
	Yes	34 (52.3%)	26 (43.3%)		
Medical Conditions/ Diseases	Diabetes	21 (32.3%)	15 (25.0%)	0.81 (1)	0.367*
	Arterial hypertension	15 (23.1%)	11 (18.3%)	0.43 (1)	0.514*
	Coronary heart disease	6 (9.2%)	8 (13.3%)	0.53 (1)	0.467*
	Chronic Heart Failure	5 (7.7%)	4 (6.7%)	0.05 (1)	1.000***
	Chronic Obstructive Pulmonary Disease	0 (0.0%)	0 (0.0%)	-	N/A
	Other	18 (27.7%)	22 (36.7%)	0.95 (1)	0.330*
Alcohol consumption in the past	No	35 (53.8%)	32 (53.3%)	0.00 (1)	0.954*
	Yes	30 (46.2%)	28 (46.7%)		
Cirrhosis etiology	Alcohol abuse	27 (41.5%)	23 (38.3%)	6.04 (8)	0.712***
	Hepatitis B/D	10 (15.4%)	10 (16.7%)		
	Hepatitis C	8 (12.3%)	7 (11.7%)		
	Non-Alcoholic Steatotic Hepatitis (NASH)	10 (15.4%)	6 (10.0%)		
	Primary Biliary Cholangitis (PBC)/ Primary sclerosing cholangitis (PSC)	5 (7.7%)	3 (5.0%)		
	Autoimmune or Cryptogenic hepatitis	3 (4.6%)	4 (6.7%)		
	Hereditary metabolic disorder	0 (0.0%)	3 (5.0%)		
	Medication	0 (0.0%)	1 (1.6%)		
	Other	2 (3.1%)	3 (5.0%)		
Daily alcohol consumption in the past (gr/24h), mean (standard deviation)		220.9 (132.2)	194.9 (159.3)	-0.68 (56)	0.550**
Time since diagnosis (years), mean (standard deviation)		6.1 (6.5)	4.7 (5.6)	-1.35 (123)	0.181**

The majority of patients in both groups were males [42 (64.6%) in the intervention group and 36 (60.0%) in the control group]. The mean ± standard deviation age of patients included in the intervention group was 66.5±11.8 years whereas in the control group was 64.2±13.7 years. The two groups had no statistically significant differences regarding the main demographic and clinical features including age, gender, education level, living conditions, professional status, income, comorbidities, medical conditions, past alcohol intake and daily consumption, time since LC diagnosis, and cirrhosis etiology (Table 1).

Adherence to medication

Data regarding patients’ adherence to overall medication as reflected by the A-14 scale questionnaire, are presented in Table 2. Medication included the following categories: diuretics, laxatives, rifamycin, norfloxacin, neomycin, beta-blockers, PEG-IFN α and/or ribavirin/ NUC, corticosteroids, ursodeoxycholic acid, folic acid, iron, magnesium, calcium, and vitamin D. Cronbach’s α for A-14 was 0.87, which was above the acceptable limit (0.7). Therefore, this tool had acceptable validity for the population of the present study.

**Table 2:** Patient adherence to medication based on A-14 scale ^†^p-values represent comparisons between the two groups in each measurement separately ^*^p-values less than 0.05 were considered statistically significant

Evaluation time	Adherence in Intervention Group (n=65)	Adherence in Control Group (n=60)	χ^2 ^(df)	p-value^†^
Baseline	50 (76.9%)	51 (85%)	1.31 (1)	0.252
3-month follow-up	65 (100%)	39 (65%)	27.34 (1)	<0.001*
6-month follow-up	57 (96.6%)	16 (28.1%)	58.4 (1)	<0.001*

At initial (baseline) evaluation, adherence rates were similar between the two groups, with no statistically significant difference (p=0.252). At 3- and 6-month follow-ups, adherence in the intervention group was significantly higher compared to the control group (p<0.001) (Table 2). Adherence in the intervention group increased at 3-month follow-up (p<0.001) and remained similar after 6 months (p=0.500) but was still higher compared to baseline (p=0.003). On the contrary, adherence rates in the control group were continuously reduced during follow-up to a statistically significant level (p<0.05 for all comparisons) (data not shown). Interestingly, according to multivariable logistic regression analysis, adherence to medication measured by A-14 scale was not associated with any independent clinical or demographic factor, including follow-up time, gender, age, education level, living conditions, employment status, family income, comorbidity, past alcohol consumption, years since diagnosis, readmission rate and number of readmissions (p>0.05).

Adherence to non-pharmaceutical recommendations

**Figure 1:**
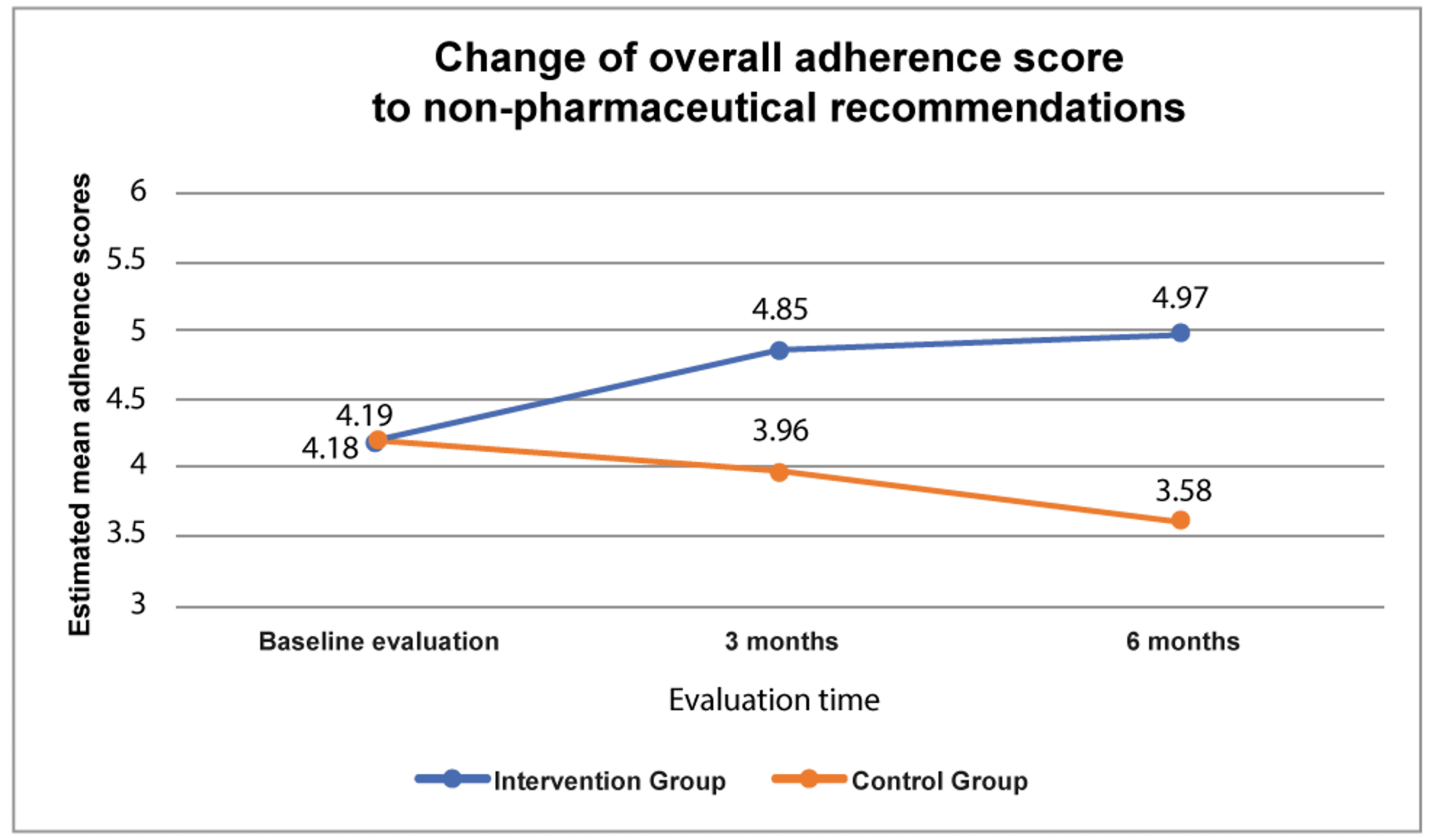
Change of overall adherence score to non-pharmaceutical recommendations

In Figure 1, the changes of overall adherence score to non-pharmaceutical recommendations are presented. Non-pharmaceutical recommendations included moderating alcohol consumption, adopting dietary modifications with attention to sodium and fat intake, maintaining up-to-date vaccinations, monitoring body weight, managing daily fluid intake, undergoing regular esophageal variceal assessments, receiving annual screenings for hepatocellular carcinoma, attending routine physician follow-ups, staying vigilant for symptom onset with guidance from medical and nursing staff, and considering the use of other medications and/or herbal supplements under professional supervision. Initially, the two groups had similar scores in all questions, with no significant difference (p>0.05). However, at 3- and 6-month follow-ups, the scores of the intervention group were statistically higher for all questions compared to the control group (p<0.001), except for alcohol consumption (p>0.05) (Figure 1). In general, adherence to non-pharmaceutical recommendations in the intervention group increased during follow-up (p <0.001), while in the control group was constantly decreased (p<0.001).

According to multivariable logistic regression analysis, adherence to non-pharmaceutical recommendations was only associated with alcohol consumption in the past. Specifically, only those patients who had consumed alcohol in the past had significantly lower adherence compared to those who had not, at initial evaluation (Table 3). On the contrary, at 3- and 6-month follow-ups, there was no statistically significant difference between adherence and past alcohol consumption (p>0.05) or any other independent clinical/demographic factor, including follow-up time, gender, age, education level, living conditions, employment status, family income, comorbidity, years since diagnosis, readmission rate, and number of readmissions (p>0.05) (data not shown).

**Table 3:** Mixed linear regression results, in a stepwise method, with adherence score to non-pharmaceutical recommendations as a dependent variable and demographic and clinical data as independent variables ^†^dependency factor, ^††^standard error ^**^p-values less than 0.05 were considered statistically significant

Demographic and Clinical Data		β^†^	SE^††^	p-value
Alcohol consumption in the past	No	N/A	N/A	N/A
	Yes	-0.42	0.18	0.022^**^
Time of adherence assessment	Baseline	N/A	N/A	N/A
	3-month follow-up	0.57	0.08	<0.001^**^
	6-month follow-up	0.43	0.09	<0.001^**^
Interaction of alcohol past consumption * Estimation time	Baseline	N/A	N/A	N/A
	3-month follow-up	0.21	0.12	0.088
	6-month follow-up	0.13	0.13	0.314

Clinical effect of patients’ adherence on quality of life, readmission rates and alcohol intake

**Figure 2:**
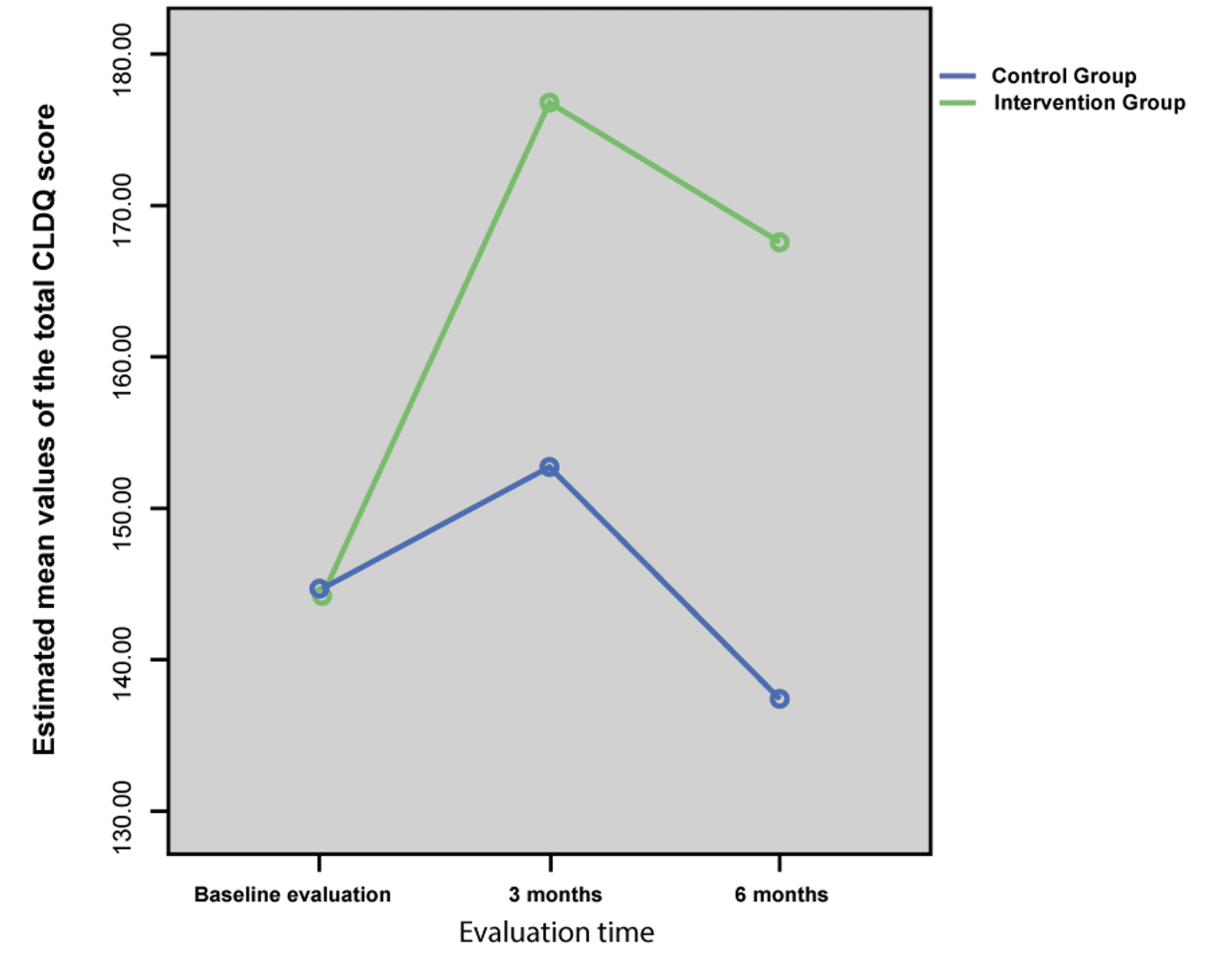
Change of mean total CLDQ score

Figure 2 shows the change of the mean total Chronic Liver Disease Questionnaire (CLDQ) score for the assessment of the patients’ QoL. According to the CLDQ questionnaire, higher scores indicate better QoL. Cronbach’s α for each CLDQ domain (fatigue, anxiety, abdominal pain, activity, emotional functionality, sleep disorders and stress) and also in overall score, was above the acceptable limit (0.7). Therefore, this tool had acceptable reliability in the study.

At initial estimation, QoL rates were similar between the two groups, with no statistically significant difference, neither in each separate domain nor totally. At 3- and 6-month follow-ups, QoL in the intervention group was significantly higher compared to the control group, both in each domain and total score (Figure 2). Moreover, QoL total score in the intervention group significantly increased after three months (p<0.001) but decreased at 6-month follow-up (p<0.001), remaining still higher compared to baseline (p<0.001). On the contrary, QoL total score in the control group increased to a significant level at 3-month follow-up (p<0.01) but decreased six months later (p<0.001), with no significant difference compared to baseline (p=0.057) (data not shown). Interestingly, according to multivariable logistic regression analysis, QoL measured by CLDQ was not associated with any independent clinical or demographic factor, including follow-up time, gender, age, education level, living conditions, employment status, family income, comorbidity, past alcohol consumption, years since diagnosis, readmission rate, and number of readmissions (p>0.05).

The proportion of patients with at least one readmission for LC-related problems six months prior to baseline, was similar between the two groups [42 (64.6%) and 37 (61.7%) in the intervention and control group respectively, p = 0.563]. After three months, the proportions were 6 (9.2%) and 6 (10.0%) respectively, with no statistically significant difference. However, six months later, the proportion of patients in the intervention group was statistically lower [12 (20.3%)] compared to the control group [28 (47.4%)], (p=0.002). Readmission rate in the intervention group was higher at baseline compared to 3- and 6-month follow-ups (p <0.001), while the readmission rate in the control group was lower at 3-month follow-up, compared to baseline and 6 months later (p<0.001). The number of readmissions in the intervention group after six months, was significantly lower compared to the control group (p=0.003). Overall, from baseline to six months later, the number of readmissions decreased in both groups (p<0.001 in the intervention group and p=0.004 in the control group).

The most common cause of readmission for both groups at baseline was ascites [22 (26.2%) for the intervention group and 19 (35%) for the control group, p=0.283]. After three months, the causes of readmissions did not differ significantly between the two groups, and six months later, the proportion of patients in the control group who were readmitted due to ascites was higher compared to the intervention group (p=0.001). The hospital stay duration (mean number of days) at baseline was significantly higher in the intervention group compared to the control group (p=0.006), while there was no statistically significant difference between the two groups during follow-up.

The proportion of patients with current alcohol consumption did not differ between the two groups during the study (p>0.05) (Table 4). Also, the proportion of patients currently consuming alcohol in the control group, increased to a statistically significant level from 3- to 6-month follow-up (p=0.031), while in the intervention group, there was no statistically significant difference during follow-up (p>0.05) (data not shown).

**Table 4:** Alcohol consumption in the present ^†^p-value for comparison between the two groups in each measurement separately (p-values less than 0.05 were considered statistically significant)

Evaluation time	Alcohol consumption in the present in Intervention Group (n=65)	Alcohol consumption in the present in Control Group (n=60)	χ^2 ^(df)	p-value^†^
Baseline	5 (7.7%)	2 (3.3%)	1.12 (1)	0.442
3-month follow-up	0 (0.0%)	1 (1.7%)	1.09 (1)	0.480
6-month follow-up	1 (1.7%)	6 (10.5%)	3.99 (1)	0.059

Correlations between readmission rates, quality of life and adherence

According to correlations between each CLDQ domain and total scores and patients’ readmissions in the intervention group at baseline, there was no statistically significant difference. However, at 3-month follow-up, patients who had not been readmitted had statistically better QoL in each separate domain and overall, compared to those who had been readmitted. The only exception was the “stress” domain, in which no statistically significant difference was found between patients who had been readmitted and those who had not.

Finally, at 6-month follow-up, patients who had not been readmitted had statistically better QoL in each separate domain and overall, compared to those who had been readmitted. The only exception was the “sleep disorders” domain, in which no statistically significant difference was found between patients who had been readmitted and those who had not.

Additionally, there was no significant difference in medication adherence in the intervention group between those patients who had been readmitted and those who had not, at baseline and at 3- and 6-month follow-ups, as well.

Table 5 shows the correlation between adherence to non-pharmaceutical recommendations in the intervention group and their readmissions at baseline, as well as at 3- and 6-month follow-ups. According to Table 5, patients who had not been readmitted at 3-month follow-up had statistically higher adherence to non-pharmaceutical recommendations compared to patients who had been readmitted (p <0.001). At initial estimation and six months later, there was no statistically significant difference (Table 5).

**Table 5:** Association of the adherence score to non-pharmaceutical recommendations with the readmissions in the intervention group ^†^p-value represents differences of adherence score to non-pharmaceutical recommendations in the intervention group between readmitted and no readmitted cases ^*^p-values less than 0.05 were considered statistically significant

Evaluation time of adherence to non-pharmaceutical recommendations	Adherence score of non-readmitted patients Mean (standard deviation)	Adherence score of readmitted patients Mean (standard deviation)	t (df)	p-value^†^
Baseline	4.13 (0.55)	4.22 (0.53)	-0.62 (61)	0.541
3-month follow-up	4.87 (0.16)	4.6 (0.28)	3.74 (62)	<0.001*
6-month follow-up	4.71 (0.28)	4.47 (0.37)	1.01 (55)	0.316

## Discussion

Liver cirrhosis is one of the leading causes of death worldwide. It is associated with serious complications in vital organs, such as anorexia, weight loss, nausea, abdominal pain, exertion, fatigue, itching, portal hypertension, esophageal varices, etc., leading to poor QoL [1]. At present, alcohol intake is the most frequent cause of liver cirrhosis in the Western world and is associated with low patient adherence. Patients affected by alcohol dependence usually have difficulty in achieving long-term total alcohol abstinence and preventing relapse. Consequently, the risk of severe consequences, including mortality increases significantly [13]. In addition, healthcare costs for decompensated cirrhosis are significantly high because these patients need intensive medical care and are often prescribed multiple medications to treat the symptoms and complications of their disease.

Despite recent advances in the management of liver disease and the treatment of viral hepatitis which may slow liver function deterioration, LC mortality is still high and the transition from compensated to decompensated cirrhosis ranges from 5-7% per year [14]. It is estimated that in patients with decompensated cirrhosis, up to 22% of readmissions within 30 days of last discharge can be avoided with better medication management or closer follow-up [15]. On average, interventional treatments related to LC patients are provided at least three times a year, requiring highly use of healthcare resources. The number of medications prescribed on discharge is an indicator of prognosis for the future readmission rate. The more the readmissions, the more the prescribed or modified medications. These data connote poor adherence, increased risk of medication errors, and a higher possibility of readmission. In existing outpatient collaboration models, proper education, good patient-provider relationship and active patient involvement in decision-making seem to improve adherence to treatment recommendations, clinic outcomes, and QoL [16, 17].

The purpose of the present study was to evaluate the effect of education on adherence to treatment recommendations and QoL in patients with LC. The mechanisms through which education impacts adherence and QoL are multifactorial. First, educational programs enhance patients' knowledge about their disease, empowering them to make informed decisions [17]. Understanding the risks of non-compliance, such as the potential for variceal bleeding or hepatocellular carcinoma (HCC), encourages patients to follow medical advice, including lifestyle modifications like alcohol cessation and regular screening for HCC [3,4]. Second, educational interventions can increase self-efficacy, or the belief in one’s ability to manage health [18]. Third, education helps reduce anxiety and fear, which are common in patients with chronic diseases like LC [19]. Finally, patient education about surveillance and disease monitoring (e.g., screening for HCC and variceal bleeding) plays a key role in reducing complications and hospitalizations [20]. In our study, patients who received education about disease monitoring showed better adherence to follow-up recommendations, which may explain the observed improvements in both treatment adherence and QoL.

Patients were randomized into two groups, intervention and control group. The intervention group received education on the disease and its treatment (a single, 30-minute educational session and a relevant leaflet), whereas the control group received the usual standard of care. The intervention group was found to have statistically higher adherence scores compared to the control group, which demonstrates the positive effect of education on adherence to medication in patients with LC. In particular, medication adherence rates, based on the A-14 scale, were similar between the two groups at baseline, with no significant difference. In this line, adherence rates in the intervention group at 3- and 6-month follow-ups were higher compared to the control group, implying a significant improvement in adherence at 3-month follow-up, which decreased at 6-month follow-up, remaining though higher than baseline levels.

No studies had been found that evaluated the effect of education on adherence to medication in patients with LC in Greece. However, international studies have shown that training patients with chronic diseases, including chronic liver disease, leads to improved medication adherence [15, 18, 21]. In the study by Volk et al. patients with LC, 25% of whom suffered from hepatic encephalopathy, received education about disease management. At baseline, only 53% of the 15 questions were answered correctly, whereas the correct response rate increased to 67% during follow-up and after education [17]. Similarly, in 2011, Larrey et al. found that frequent education by nurses in patients with chronic hepatitis C treated with interferon and ribavirin led to higher medication adherence rates and sustained virologic response (SVR), compared to those who had received the usual standard of care (69.7% vs. 53.3%, p <0.05 and 38.2% vs. 24.8%, respectively) [22].

It is worth noting that in most studies the correlation between patients’ demographic and/or clinical characteristics and the effect of education on medication adherence has not been evaluated. Factors such as age, gender, disease severity, comorbidities, education level, availability of a support network, depression, and medication complexity can independently affect medication adherence. In our study, the improvement of adherence in the intervention group was not found to be statistically associated with demographic and clinical data, indicating that the effect of education was independent of several confounding factors mentioned previously.

Successful LC treatment is also associated with the adoption and maintenance of certain lifestyle modifications [22]. Thus, a 10-point Likert scale created by the researchers was used to evaluate patient adherence to non-pharmaceutical recommendations. Adherence to lifestyle changes in patients with LC was evaluated in the following domains: alcohol consumption, diet, vaccination, body weight monitoring, daily fluid intake, esophageal varices monitoring, annual examination for hepatocellular carcinoma, regular meetings with healthcare professionals, timely reporting of symptom onset, and informing healthcare professionals before taking new medications or herbal supplements. According to this scale, patient adherence to non-pharmaceutical recommendations was similar between the two groups at baseline. At 3- and 6-month follow-ups, patient adherence in the intervention group was statistically higher compared to the control group. Over time, adherence in the intervention group increased, while adherence in the control group continuously decreased. Therefore, education appeared to have a positive effect on patient adherence to non-pharmaceutical recommendations. None of the demographic and clinical variables were found to be statistically associated with adherence improvement in the intervention group, except history of alcohol consumption.

In 2019, Cui et al. conducted a study on hospitalized patients with chronic hepatitis B or C, some of whom had LC. Some patients received two in-person training sessions by specialized nurses as well as monthly telephone counseling at home after their discharge, while the rest received the usual standard of health care (controls). Data collected during baseline and six months later showed that there was adherence improvement to non-pharmaceutical recommendations, such as restriction of alcohol intake, dietary habits, and stress management in the trained group [23]. Furthermore, Singal et al. found that patients with LC who were informed about their disease and felt more involved in their care were more likely to adhere to non-pharmaceutical recommendations and especially to annual screening for hepatocellular carcinoma [20].

Patients with LC often experience complications including ascites, gastrointestinal bleeding and infections, as well as restrictions in personal and social relationships, affecting adversely their physical and emotional status and therefore, their quality of life [18, 24, 25]. Nursing interventions, such as education are essential in improving these patients’ QoL [24, 25].

In the present study, patients in the intervention group had similar CLDQ scores to the control group, at baseline. After training, the intervention group had statistically better QoL compared to the control group, across all CLDQ domains. According to the multifactorial statistical analysis, the rate of change in the trained patients’ QoL, was not found to be statistically associated with any demographic or clinical factor, as shown by other studies as well [24, 25]. Therefore, education seems to have independently contributed to the improvement of patients' QoL.

Similarly, Sharif et al. used the CLDQ questionnaire to assess the QoL in patients with chronic liver disease. Patients initially received training through three individual sessions, followed by a group session, while the control group received the usual standard of care. The comparison of QoL between the two groups at baseline and one day later showed a significant difference in only two domains, fatigue and emotional function. However, at the 3-month follow-up, significant differences were found in all domains of CLDQ between the two groups. These findings suggest that education had a positive impact on all aspects of QoL [19].

In 2015, Taha et al. also used the CLDQ questionnaire to assess the QoL in patients with LC. The intervention group received training from specialized nurses. After training, patients in the intervention group had higher scores in three CLDQ domains (activity, emotion and anxiety) compared to the control group. At the 2-month follow-up QoL in the intervention group improved in the following domains: abdominal symptoms, fatigue, and systemic symptoms [24].

Cirrhotic patients have increased readmission rates for LC-related health problems. In a recent study, more than half (53%) of patients with decompensated LC were readmitted within three months after discharge [26]. Similar studies have shown that readmission occurs even earlier (≤30 days after discharge) in 20-37% of patients with LC [27, 28]. Additionally, early readmissions in combination with liver disease severity have been associated with higher mortality [28].

In the present study, the proportion of patients in the intervention group who reported at least one readmission was statistically lower compared to the control group at 6-month follow-up. Thus, it seems that training contributed to reduce patient readmissions, apparently due to better disease management. Other studies in the international literature are in line with this finding. Specifically, in the study of Morales et al., one group of patients participated in the HEPACONTROL training program while the control group did not receive any training after discharge. As a result, the intervention group had lower readmission rates compared to the control group (11.3% vs. 29.5%) [27]. In contrast, the study of Kanwal et al. showed no positive effect of education on reducing the number of readmissions [29].

In this study, statistical analysis showed that patients in the intervention group who were not readmitted at 3-month follow-up had statistically higher adherence to non-pharmaceutical recommendations compared to the control group. Higher adherence to treatment (both for medications and non-pharmaceutical recommendations) obviously leads to better disease management and subsequently, fewer readmissions. Similarly, Koelling et al. found that patients with heart failure who participated in a disease training program and reported that they weighed themselves daily and reduced sodium intake during the 30-day follow-up, had a lower readmission rate compared to the control group [30].

In our study, it was also found that patients in the intervention group who were not readmitted at 3- and 6-month follow-ups had statistically better QoL overall, compared to those who were readmitted. Thus, it seems that reduced readmissions during follow-up are associated with better disease management and improved physical well-being.

Inevitably, the current study has some limitations. First, the study was conducted in a single center. Multicenter studies would be expected to ensure larger samples of LC patients and provide further insights into this field. Second, the sample size was relatively small, since patients were recruited through convenience sampling, which may limit the generalizability of the results and the exclusion criteria were very stringent. Third, the educational intervention was performed only once at baseline. The positive effects of the intervention, as measured by specific tools (e.g., Morisky Medication Adherence Scale, SF-36 Health Survey, etc.), are expected to be further augmented by additional educational sessions over time and on a regular basis. Fourth, patients with severe cirrhosis and Grade III or IV hepatic encephalopathy/Child-Pugh stage C, who might benefit from the educational intervention, were excluded due to their inability to cooperate. Fifth, we collected no information regarding the presence of ascites at initial presentation or during hospitalization which could have potentially provided more insights into the effect of educational intervention on LC patients. Finally, apart from a nurse, no other healthcare professionals were involved in the educational process, which could have potentially improved the impact of education.

## Conclusions

The current study showed that education significantly improved patient adherence and QoL, leading to better disease management. No demographic or clinical factors were found to be statistically relevant to the improvement of adherence and QoL, which demonstrates the independent effect of education on patient outcomes and QoL. In addition, reduced readmissions in the intervention group during follow-up were associated with better disease management and improved physical well-being. More studies with larger sample sizes and longer follow-up periods, including additional educational interventions as part of routine care for patients with LC are anticipated to reform recommendations for integrating educational interventions into standard care to improve QoL and disease outcomes.

These interventions should include regular educational sessions delivered by trained healthcare professionals during clinical visits, with content tailored to the individual patient’s needs, such as medication management, lifestyle changes, and symptom monitoring. A multidisciplinary approach involving dietitians, hepatologists, and psychologists could provide a more holistic treatment plan, addressing both physical and mental health aspects. Additionally, using technology like mobile apps, online resources, and telemedicine can enhance patient engagement and support ongoing education. Regular follow-up assessments of patient adherence, knowledge, and QoL are essential for tracking progress and refining educational content to meet patients' evolving needs. Ongoing assessment of adherence, efficacy, and education enhancement is a crucial area of future research for improving long-term management in patients with LC.

## Appendices

Appendix A

Adherence to Non-Pharmaceutical Recommendations Assessment Tool for Patients with Liver Cirrhosis

There is no validated tool to assess adherence to non-pharmaceutical recommendations in patients with liver cirrhosis. For this reason, the researchers created a new, specialized assessment tool, based on international guidelines of the American College of Gastroenterology (ACG) and the European Association for the Study of the Liver (EASL) [1-4], further supported by international bibliography [5-8]. This 10-item Likert scale demonstrated reliability and validity for use in the Greek population (Cronbach’s α = 0.79). The overall score ranges from 10 to 50, with higher scores indicating greater adherence.

Appendix B

Educational Leaflet: "What Do I Need to Know about Liver Cirrhosis?"

The educational leaflet was designed to inform patients with liver cirrhosis about the disease’s pathophysiology, symptoms and treatment methods. It was specifically created by the researchers to meet the needs of the present study. The content included was sourced from recent, relevant literature [1, 5-8].
